# Increased Levels of Oxidative Stress in Human Prostate Intraepithelial Neoplasia and Prostate Cancer: Evidence from 4-Hydroxyneonal Detection and Its Implications

**DOI:** 10.3390/antiox14091060

**Published:** 2025-08-28

**Authors:** Geou-Yarh Liou, Woojung Kim, Tamiya M. Hobbs

**Affiliations:** 1 Center for Cancer Research and Therapeutic Development, Clark Atlanta University, Atlanta, GA 30314, USA; 2 Department of Biological Sciences, Clark Atlanta University, Atlanta, GA 30314, USA

**Keywords:** oxidative stress, reactive oxygen species, 4HNE protein adducts, prostate intraepithelial neoplasia, prostate cancer, inflammation

## Abstract

Prostate cancer is not only the most common type of cancer in elderly American men but also the 2nd leading cause of cancer death in American men. The currently available treatments in clinics target male hormones that are majorly required for maintaining many physiological functions, including muscle strength, leading to poor life quality and subsequent patient-opted intermittent treatment. Aging is a key factor in prostate cancer that is associated with increased levels of oxidative stress. Several lines of evidence indicated elevated levels of reactive oxygen species (ROS) in prostate cancer, including its precursor, prostate intraepithelial neoplasia (PIN). In this current study, we utilized 4-hydroxynonenal (4HNE) as a general readout for overall oxidative stress to demonstrate the imbalance between ROS and antioxidants in human prostate cancer and its precursor lesion in both human culture cell lines and tissue samples. Our results showed that the production of 4HNE adducts was increased in human prostate cancer cells and was non-linearly correlated with prostate cancer stage. They also provided insight into prevention and potential therapeutic strategies for prostate cancer.

## 1. Introduction

Oxidative stress is an imbalance between ROS and antioxidants, typically with a high amount of reactive oxygen species and a weakened antioxidant defense system or insufficient antioxidants present in the system. Elevated levels of ROS from oxidative stress have been linked to almost all disorders, including prostate cancer [1,2,3]. Prostate cancer is highly prevalent in elderly American men whose age is 65 or older, and its incidence is on a constant rise annually [4,5]. Although the majority of prostate cancer patients are diagnosed at an early stage, still it is the second leading cause of cancer death in American men [4,5] without a sign of slowing down its death toll. Among all known risk factors, including family history, genetics, ethnicity, etc., aging is the key factor for prostate cancer. Aging is tightly associated with elevated oxidative stress. During the aging process, more highly reactive oxygen species (ROS) are produced, and these excessive, un-neutralized ROS further react with DNA, proteins, and lipids to cause damage such as DNA strand breaks, DNA adducts, lipid peroxidation, etc., at all levels, thus leading to cellular dysfunctions [1,6].

While it remains challenging to detect all reactive oxygen species that have very short half-lives, other oxidative stress-induced major biomolecules have been researched as an alternative for the biomarker for elevated oxidative stress. One of the most widely recognized biomolecules is 4-hydroxynonenal (4HNE). 4HNE is a highly reactive aldehyde majorly generated during the process of oxidative degradation of polyunsaturated fatty acids in cell membranes in response to elevated oxidative stress [7,8]. Besides lipids [9,10,11], 4HNE also can directly react with proteinsand nucleic acids [12,13] to form stable 4HNE adducts, which are critical in cellular signaling as a second messenger and other cellular processes, e.g., apoptosis or glucose transport [8,14]. Herein we showed that elevated levels of 4HNE protein adducts were present in human prostate cancer cell lines, including LNCaP, Du145 and PC3. Furthermore, more 4HNE adduct formation was present in human prostate cancer tissues and precancerous lesion of the prostate, PIN. The detected 4HNE levels of the tissue samples were not linearly dependent on prostate cancer stage. Our findings not only confirm the high oxidative stress status in prostate cancer using 4HNE, the most crucial oxidative stress marker for a broad imbalance between ROS and antioxidants, but also provide insight into prevention and possible new therapy strategies.

## 2. Materials and Methods

### 2.1. Cell Lines, Antibodies and Reagents

All cell lines used in this manuscript were purchased from ATCC (Manassas, VA, USA). Murine macrophage Raw 264.7 cells and human prostate cancer LNCaP cells were cultured in RPMI-1640 containing 100 U/mL penicillin/streptomycin and 10% FBS. Similarly, human prostate cancer Du-145 cells were cultured in high-glucose DMEM media supplemented with 100 U/mL penicillin/streptomycin and 10% FBS. Human normal prostate epithelial cells PZ-HPV7 were cultured as previously described [15]. In brief, PZ-HPV7 cells were cultured in Keratinocyte Serum-Free media containing 0.05 mg/mL bovine pituitary extract and 5 ng/mL human recombinant EGF. All cell lines were from ATCC (Manassas, VA, USA). Cells were maintained in a humidified 37 °C incubator with 5% CO_2_.

4NHE antiserum was purchased from Alpha Diagnostic International (San Antonio, TX, USA). GAPDH antibody was from Cell Signaling Technology (Danvers, MA, USA). Βeta-actin antibody was from Santa Cruz Biotechnology (Dallas, TX, USA). Other reagents used are described in the specific experiment sections.

### 2.2. Generation of Macrophage-Conditioned Media and Treatment of PZ-HPV7 Cells

To obtain macrophage-conditioned media, 5 × 10^5^ Raw264.7 cells per 6-well dish were grown in Keratinocyte Serum-Free complete media, and supernatant was collected. The freshly generated macrophage-conditioned media was then incubated with PZ-HPV7 cells for 48 h before harvesting total cell lysates.

### 2.3. Total Cell Lysates Collection and Immunoblotting

Cells were rinsed with cold PBS twice, lysed in buffer A (50 mM Tris/HCl [pH 7.4], 1% TritonX-100, 5 mM EDTA [pH 7.4] and 150 mM NaCl) or RIPA buffer (25 mM Tris/HCl [pH 7.5], 1% Triton X-100, 140 mM NaCl, 1 mM EDTA, 0.5% SDS) containing protease inhibitor cocktail (Thermo Fisher Scientific (Waltham, MA, USA)), vortexed at the maximal speed for 1 min, incubated at 4 °C for 10 min, and centrifuged at the same temperature at 14,000 rpm for 10 min. The supernatants were denatured and subjected to SDS-PAGE. Resolved proteins were transferred to a nitrocellulose membrane prior to blocking in 5% BSA in TBST (50 mM Tris.HCl [pH 7.6], 0.05% Tween 20, 150 mM NaCl) and incubated with the antibodies of interest in 5% BSA in TBST overnight at 4 °C. The appropriate horseradish peroxidase-conjugated secondary antibodies were added to the membranes for 30 min at room temperature and then processed with 3 TBST washes. Images were captured and visualized with ECL and X-ray film.

### 2.4. Human Prostate Tissue Samples

De-identified human prostate tissue samples, including normal, prostatic intraepithelial neoplasia (PIN), and cancer, were obtained from BioChain (Newark, CA, USA), TissueArray.com (Derwood, MD, USA), and Cooperative Human Tissue Network (CHTN). All experiments involved in the use of human tissue samples are carried out according to the IRB protocol approved by the CAU IRB committee.

### 2.5. Immunohistochemistry and Quantification

The procedure has been described previously [16,17]. In brief, slides were deparaffinized in xylene and gradually re-hydrated through 100% alcohol to distilled water. The rehydrated slides were subjected to heat-induced antigen retrieval in the antigen retrieval buffer, 10 mM sodium citrate buffer (pH 6.0). Slides were incubated with 3% hydrogen peroxide to decrease endogenous peroxidase activity and rinsed with PBS. Slides were treated with protein block serum free solution (DAKO) for 10 min at room temperature. After the 4HNE antibody at 1:500 dilution was incubated with slides, the ImmPRESS Polymer Detection Kit (Vector Laboratories (Burlingame, CA, USA)) was used according to the manufacturer’s instructions to visualize stained slides. Images were collected using the Aperio VERSA tissue scanner with ImageScope software 12.4.3 (Aperio (Sausalito, CA, USA)). To quantify the 4HNE intensity in each tissue sample, ImageJ 1.54 was used to measure the integrated intensity of each local area. Randomly selected five independent local areas in each tissue sample were used for analysis.

### 2.6. Statistical Analysis

All results shown are representative of 3 independent experiments or tissue samples. Data are presented as ± SE, while *p*-values were acquired using Student’s *t*-test for comparing 2 sets of data using Prism (GraphPad Software 7 (San Diego, CA, USA)). Data sets with more than 2 sets of data were analyzed using one-way ANOVA, along with multiple comparisons carried out using Prism (GradPad Software). *p* < 0.05 is considered statistically significant.

## 3. Results

### 3.1. Macrophage-Conditioned Media Increased Formation of 4HNE Protein Adducts in Immortalized Human Normal Prostate Epithelial Cell Line PZ-HPV7

**Figure 1:**
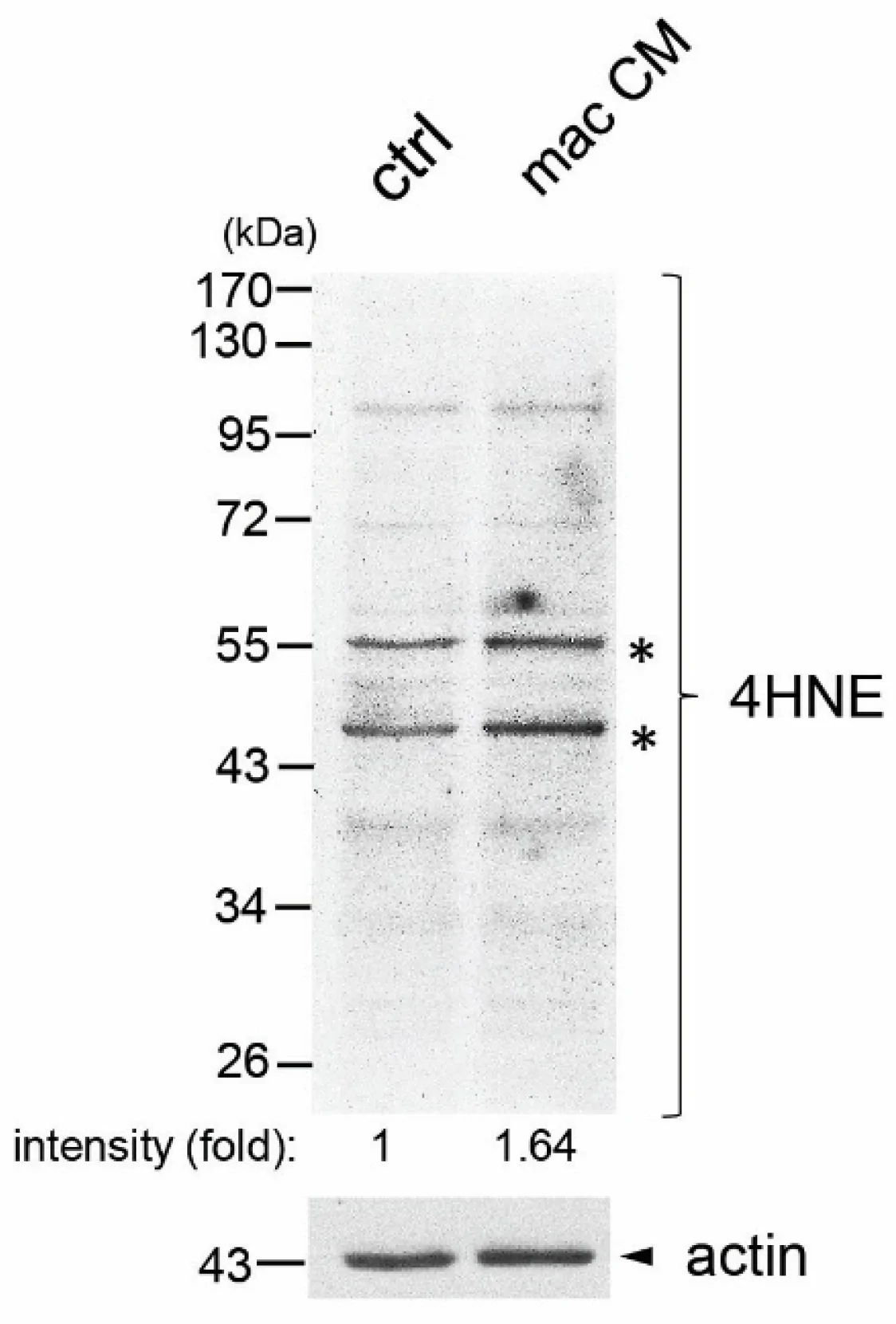
Increased levels of 4HNE protein adducts in human PZ-HPV7 prostate epithelial cells in response to macrophage-conditioned media treatment. PZ-HPV7 cells were treated with either control media or macrophage-conditioned media for 48 h. Total cell lysates were collected and subjected to immunoblotting for 4HNE and actin. The relative intensity fold change for the indicated bands (labeled with asterisks) was calculated by the ratio of the levels of 4HNE protein adducts over the actin protein levels in each condition and set as 1 for control treatment. Each image shown was representative of 3 independent experiments.

Since macrophages are one of the major sources of oxidative stress via producing reactive oxygen species (ROS) during inflammation and in response to pathogens, we then treated human normal prostate epithelial cells PZ-HPV7 with or without macrophage Raw264.7 cell-derived conditioned media for 48 h and used this conditioned-media treatment as a positive control for 4HNE evaluation via immunoblots. As shown in Figure 1, there were several bands ranging from different molecular weights detected as formed 4HNE protein adducts in PZ-HPV7 normal prostate epithelial cells. Furthermore, when treating with the macrophage-conditioned media, two 4HNE-formed protein adducts with a molecular weight of around 55 and 45 kDa were elevated. This indicated 4HNE protein adduct formation detected by 4HNE immunoblotting can be used as a readout for oxidative stress and a general production of reactive oxygen species (ROS).

### 3.2. Elevated Production of 4HNE Protein Adducts in Human Prostate Cancer Cell Lines

**Figure 2:**
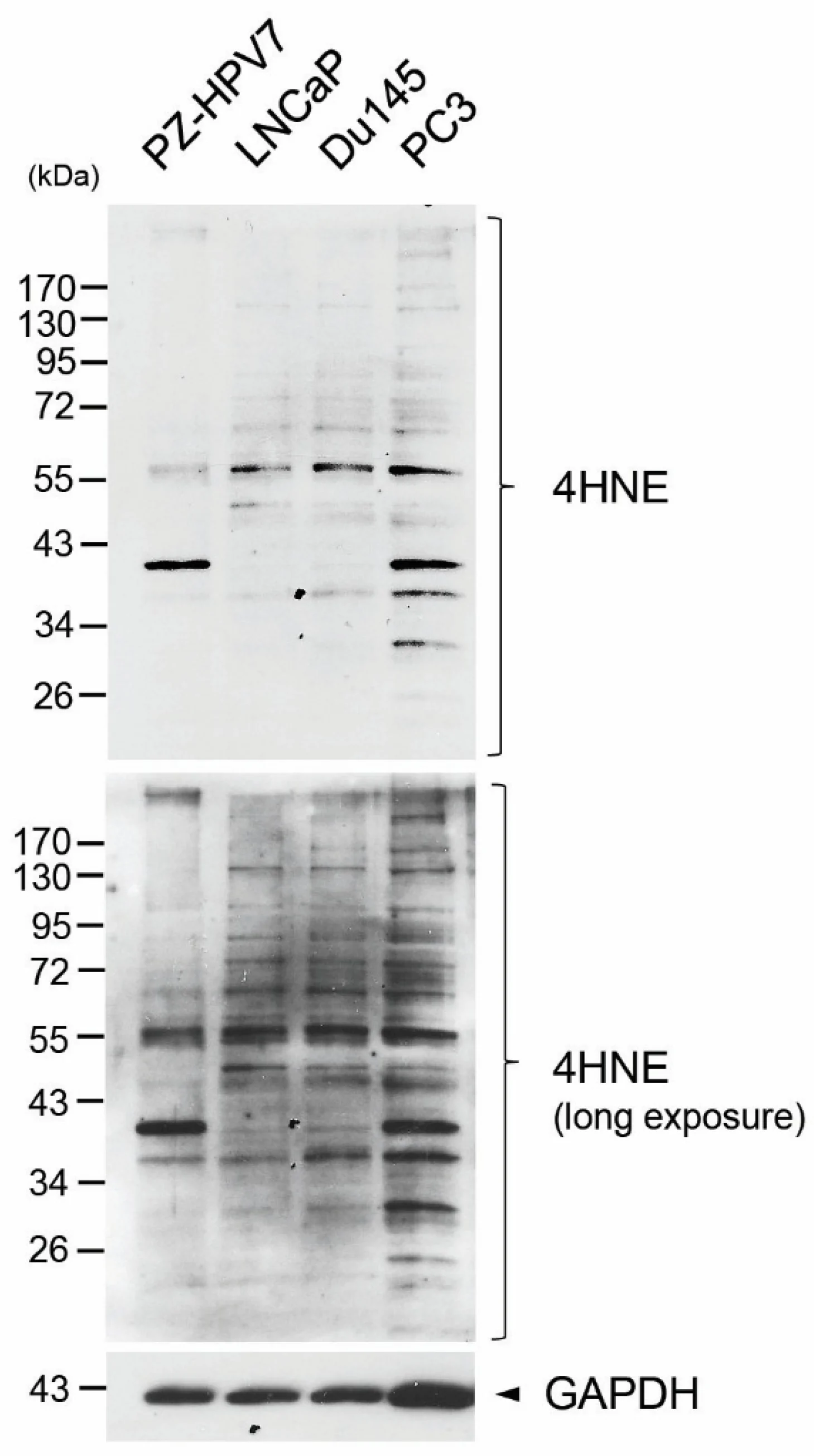
Levels of 4HNE protein adducts in human prostate cancer cells and normal prostate epithelial cells. Total cell lysates collected from human prostate cancer cell lines, including LNCaP, Du145, and PC3, as well as human normal epithelial prostate cells PZ-HPV7, were examined for levels of 4HNE protein adducts via 4HNE immunoblotting. GAPDH was used as a loading control. Each image shown was representative of 3 independent experiments.

To assess whether human prostate cancer cells have higher levels of oxidative stress, we subjected total cell lysates of the human normal prostate epithelial cell line PZ-HPV7 and human prostate cancer cell lines, including LNCaP, Du145, and PC3, to 4HNE immunoblotting. As shown in Figure 2, there were significantly more formed 4HNE protein adducts in all prostate cancer cells, LNCaP, Du145, and PC3, as compared to PZ-HPV7 normal prostate epithelial cells, suggesting that human prostate cancer cells possess higher levels of oxidative stress. Furthermore, one of the major 4HNE protein adducts with a molecular weight of around 55 kDa was increased in all 3 prostate cancer cell lines regardless of the status of androgen receptor among them. Some of the minor 4HNE protein adducts, such as those around 38 kDa, 30 kDa, or a higher molecular weight, were also increased in prostate cancer cells. Altogether, these suggest that prostate cancer cells, regardless of their AR status, do have elevated oxidative stress in comparison to normal prostate epithelial cells.

### 3.3. More Formations of 4HNE Adducts Were Present in Human Tissues of PIN and Prostate Cancer

**Figure 3:**
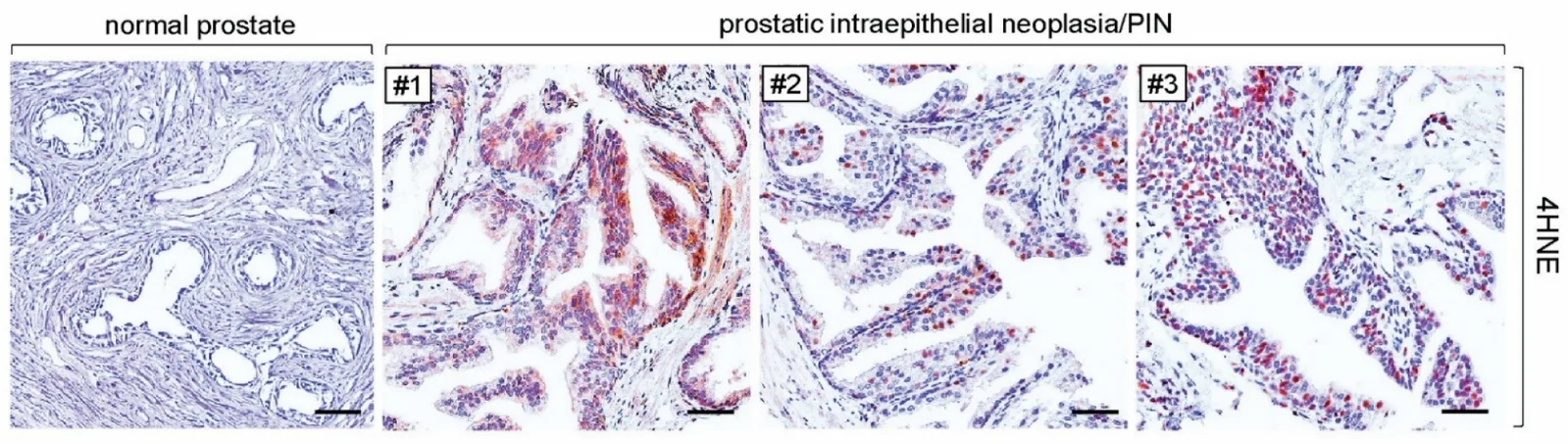
Increased 4HNE adducts were present in human PIN cells. Human tissue samples of PIN and normal prostate were immunostained with the 4HNE antiserum to assess their levels of oxidative stress. *n* = 15, scale bar: 50 µm.

**Figure 4:**
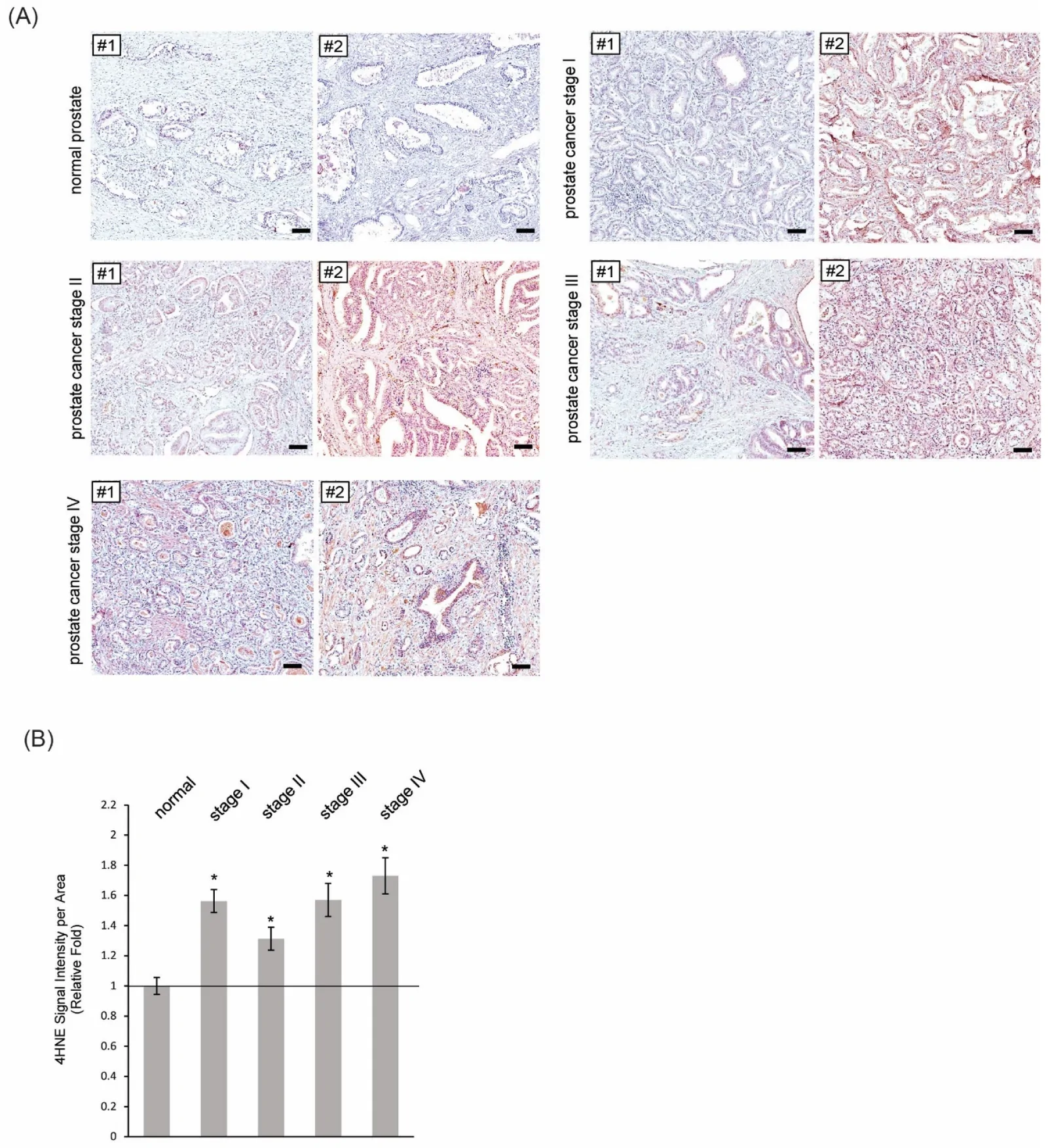
Levels of 4HNE adducts in different stages of human prostate cancer. (**A**) Human prostate cancer tissues collected at the indicated stages were subjected to 4HNE immunohistochemistry for examining their oxidative stress levels using 4HNE as a readout. Human normal prostate tissue from individuals without prostate disorders was used as a control. *n* = 5, scale bar: 100 µm. (**B**) The 4HNE staining intensity in each tissue sample shown in (**A**) was quantified and graphed according to the cancer stage. The solid line indicated a relative fold of 1 to the normal prostate tissues. *: *p* < 0.05 as compared to the human normal prostate tissues.

Prostate intraepithelial neoplasia (PIN) is a precursor of prostate cancer and is associated with cellular proliferations of prostate epithelial cells within preexisting ducts and acini. Accumulating evidence [18,19,20] showed chronic inflammation promotes development and progression of PIN, thus increasing the chance for prostate cancer onset. To assess the oxidative stress levels in human PIN, human prostate tissue samples that contain PIN structures were subjected to immunohistochemistry using the 4HNE anti-sera. As shown in Figure 3, increased formations of 4HNE adducts were present in human PIN regions as compared to normal human prostate tissues. We next assessed the oxidative stress levels in human prostate cancer tissue samples that were categorized into stages I to IV. Human prostate cancer samples, regardless of their stage, had a higher production of 4HNE adducts as compared to normal prostate tissues (Figure 4A). In addition, there was no proportional correlation between the amount of 4HNE adducts formed and prostate cancer stage (Figure 4B), indicating no linear correlation between the levels of oxidative stress and prostate cancer stage.

## 4. Discussion

Oxidative stress is an imbalance between ROS and antioxidants and has been linked to almost all human disorders, including prostate cancer. In cultured human prostate cancer cell lines such as LNCaP, Du145, and PC3 cells, increased transcripts of NOX enzymes that produce cellular ROS were detected [21]. Furthermore, treatment with the NOX inhibitor diphenyleneiodonium attenuated cancer cell proliferation, migration, and invasiveness. Mitochondria is the major source to produce cellular ROS, and several reports demonstrated increased mitochondrial activity via higher expression levels and activity of mitochondrial glycerophosphate dehydrogenase [22,23], downregulation of mitochondrial creatine kinase [24], higher succinate oxidation with mtDNA mutations [25] and increased uptake of lactate that led to PGC1α activation [26]. All these mechanisms described above result in higher ROS production in human prostate cancer cells, fueling disease progression and cancer cell aggressiveness.

Reduced expression levels of antioxidants such as catalase, mitochondrial manganese superoxide dismutase (MnSOD), CuZnSOD, and reduced glutathione (GSH), not surprisingly, have been shown in human prostate cancer patient specimens, including tissue samples [27,28,29]. However, others reported opposite results for these antioxidant enzymes [23,30,31]. Other antioxidant proteins, such as peroxiredoxin 4, were also shown to be highly expressed in prostate cancer patient specimens and cultured prostate cancer cell lines [32] and promoted prostate cancer growth and survival. Our results, as shown in Figure 2 and Figure 4, clearly indicated a net increase in ROS, known as increased oxidative stress, in human prostate cancer tissues through detection of 4HNE adduct formations, and these adducts can form at levels of nucleic acids, proteins, or lipids. In addition, our results (Figure 4) showed that regardless of cancer stage, the 4HNE levels were significantly elevated in prostate cancer specimens by two-fold as compared to the normal prostate tissue samples. This is very likely because the moderately increased levels of ROS that are within a threshold do promote cancer cell proliferation, cell migration, and cell abilities to invade and transform, while either extremely high or low levels of ROS induce apoptosis. To apply our findings to prevention, intervention, and/or therapy of prostate cancer in elderly American men, a decrease in oxidative stress by either elevating or reducing ROS antioxidants (through lifestyle, diet, moderate exercise, etc.) may help prevent the development of prostate cancer as well as slow down the progression of prostate cancer at an early stage. For late-stage prostate cancer, it would be more beneficial to dramatically enhance oxidative stress to an extremely high level through therapeutic approaches to induce cell death of prostate cancer cells.

Among all ethnicity groups, African American men have the highest incident rate and the highest mortality rate of prostate cancer [33,34,35]. Accumulating evidence showed higher oxidative stress and inflammatory body conditions in African Americans [36,37,38,39] which provides a possible rationale for prostate cancer disparity in this racial group. Given that prostate cancer is heavily influenced by the tumor environment, which can be altered through diet and lifestyle, how to constantly diminish oxidative stress remains crucial to prevent prostate cancer or keep early stages of prostate cancer at bay.

## 5. Conclusions

4HNE is a well-recognized biomarker for oxidative stress. Using 4HNE as a readout for enhanced oxidative stress, human prostate cancer cells and its precursor lesion PIN do have elevated oxidative stress. For the high-risk population for prostate cancer or those who are diagnosed with an early stage of prostate cancer, targeting oxidative stress through lifestyle, diet, exercise, and/or medicine can be one of the strategies to prevent or slow down prostate cancer growth.

## References

[1] Liou G.Y., Storz P. (2010). Reactive oxygen species in cancer. Free Radic. Res..

[2] Dubois-Deruy E., Peugnet V., Turkieh A., Pinet F. (2020). Oxidative Stress in Cardiovascular Diseases. Antioxidants.

[3] Kim G.H., Kim J.E., Rhie S.J., Yoon S. (2015). The Role of Oxidative Stress in Neurodegenerative Diseases. Exp. Neurobiol..

[4] Nierengarten M.B. (2024). Cancer Statistics 2024: Deaths drop, incidences increase, prevention needed. Cancer.

[5] Siegel R.L., Giaquinto A.N., Jemal A. (2024). Cancer statistics, 2024. CA Cancer J. Clin..

[6] Juan C.A., Perez de la Lastra J.M., Plou F.J., Perez-Lebena E. (2021). The Chemistry of Reactive Oxygen Species (ROS) Revisited: Outlining Their Role in Biological Macromolecules (DNA, Lipids and Proteins) and Induced Pathologies. Int. J. Mol. Sci..

[7] Catala A. (2009). Lipid peroxidation of membrane phospholipids generates hydroxy-alkenals and oxidized phospholipids active in physiological and/or pathological conditions. Chem. Phys. Lipids.

[8] Dalleau S., Baradat M., Gueraud F., Huc L. (2013). Cell death and diseases related to oxidative stress: 4-hydroxynonenal (HNE) in the balance. Cell Death Differ..

[9] Breitzig M., Bhimineni C., Lockey R., Kolliputi N. (2016). 4-Hydroxy-2-nonenal: A critical target in oxidative stress?. Am. J. Physiol. Cell Physiol..

[10] Mihalas B.P., De Iuliis G.N., Redgrove K.A., McLaughlin E.A., Nixon B. (2017). The lipid peroxidation product 4-hydroxynonenal contributes to oxidative stress-mediated deterioration of the ageing oocyte. Sci. Rep..

[11] Zhong H., Yin H. (2015). Role of lipid peroxidation derived 4-hydroxynonenal (4-HNE) in cancer: Focusing on mitochondria. Redox Biol..

[12] Ji Y., Dai Z., Wu G., Wu Z. (2016). 4-Hydroxy-2-nonenal induces apoptosis by activating ERK1/2 signaling and depleting intracellular glutathione in intestinal epithelial cells. Sci. Rep..

[13] Shoeb M., Ansari N.H., Srivastava S.K., Ramana K.V. (2014). 4-Hydroxynonenal in the pathogenesis and progression of human diseases. Curr. Med. Chem..

[14] Riahi Y., Cohen G., Shamni O., Sasson S. (2010). Signaling and cytotoxic functions of 4-hydroxyalkenals. Am. J. Physiol. Endocrinol. Metab..

[15] Dang T., Liou G.Y. (2018). Macrophage Cytokines Enhance Cell Proliferation of Normal Prostate Epithelial Cells through Activation of ERK and Akt. Sci. Rep..

[16] Liou G.Y., Byrd C.J., Storz P., Messex J.K. (2024). Cytokine CCL9 Mediates Oncogenic KRAS-Induced Pancreatic Acinar-to-Ductal Metaplasia by Promoting Reactive Oxygen Species and Metalloproteinases. Int. J. Mol. Sci..

[17] Messex J.K., Adams K.L.A., Hawkins W.G., DeNardo D., Bardeesy N., Billadeau D.D., Liou G.Y. (2022). Oncogenic Kras-Mediated Cytokine CCL15 Regulates Pancreatic Cancer Cell Migration and Invasion through ROS. Cancers.

[18] Messex J.K., Byrd C.J., Thomas M.U., Liou G.Y. (2022). Macrophages Cytokine Spp1 Increases Growth of Prostate Intraepithelial Neoplasia to Promote Prostate Tumor Progression. Int. J. Mol. Sci..

[19] Thomas M.U., Messex J.K., Dang T., Abdulkadir S.A., Jorcyk C.L., Liou G.Y. (2021). Macrophages expedite cell proliferation of prostate intraepithelial neoplasia through their downstream target ERK. FEBS J..

[20] Elkahwaji J.E., Hauke R.J., Brawner C.M. (2009). Chronic bacterial inflammation induces prostatic intraepithelial neoplasia in mouse prostate. Br. J. Cancer.

[21] Kumar B., Koul S., Khandrika L., Meacham R.B., Koul H.K. (2008). Oxidative stress is inherent in prostate cancer cells and is required for aggressive phenotype. Cancer Res..

[22] Chowdhury S.K., Gemin A., Singh G. (2005). High activity of mitochondrial glycerophosphate dehydrogenase and glycerophosphate-dependent ROS production in prostate cancer cell lines. Biochem. Biophys. Res. Commun..

[23] Chowdhury S.K., Raha S., Tarnopolsky M.A., Singh G. (2007). Increased expression of mitochondrial glycerophosphate dehydrogenase and antioxidant enzymes in prostate cancer cell lines/cancer. Free Radic. Res..

[24] Amamoto R., Uchiumi T., Yagi M., Monji K., Song Y., Oda Y., Shiota M., Yokomizo A., Naito S., Kang D. (2016). The Expression of Ubiquitous Mitochondrial Creatine Kinase Is Downregulated as Prostate Cancer Progression. J. Cancer.

[25] Schopf B., Weissensteiner H., Schafer G., Fazzini F., Charoentong P., Naschberger A., Rupp B., Fendt L., Bukur V., Giese I. (2020). OXPHOS remodeling in high-grade prostate cancer involves mtDNA mutations and increased succinate oxidation. Nat. Commun..

[26] Ippolito L., Morandi A., Taddei M.L., Parri M., Comito G., Iscaro A., Raspollini M.R., Magherini F., Rapizzi E., Masquelier J. (2019). Cancer-associated fibroblasts promote prostate cancer malignancy via metabolic rewiring and mitochondrial transfer. Oncogene.

[27] Arsova-Sarafinovska Z., Eken A., Matevska N., Erdem O., Sayal A., Savaser A., Banev S., Petrovski D., Dzikova S., Georgiev V. (2009). Increased oxidative/nitrosative stress and decreased antioxidant enzyme activities in prostate cancer. Clin. Biochem..

[28] Baker A.M., Oberley L.W., Cohen M.B. (1997). Expression of antioxidant enzymes in human prostatic adenocarcinoma. Prostate.

[29] Bostwick D.G., Alexander E.E., Singh R., Shan A., Qian J., Santella R.M., Oberley L.W., Yan T., Zhong W., Jiang X. (2000). Antioxidant enzyme expression and reactive oxygen species damage in prostatic intraepithelial neoplasia and cancer. Cancer.

[30] Chaiswing L., Zhong W., Oberley T.D. (2014). Increasing discordant antioxidant protein levels and enzymatic activities contribute to increasing redox imbalance observed during human prostate cancer progression. Free Radic. Biol. Med..

[31] Li N., Oberley T.D., Oberley L.W., Zhong W. (1998). Overexpression of manganese superoxide dismutase in DU145 human prostate carcinoma cells has multiple effects on cell phenotype. Prostate.

[32] Ding N., Jiang H., Thapa P., Hao Y., Alshahrani A., Allison D., Izumi T., Rangnekar V.M., Liu X., Wei Q. (2022). Peroxiredoxin IV plays a critical role in cancer cell growth and radioresistance through the activation of the Akt/GSK3 signaling pathways. J. Biol. Chem..

[33] Chowdhury-Paulino I.M., Ericsson C., Vince R., Spratt D.E., George D.J., Mucci L.A. (2022). Racial disparities in prostate cancer among black men: Epidemiology and outcomes. Prostate Cancer Prostatic Dis..

[34] Hinata N., Fujisawa M. (2022). Racial Differences in Prostate Cancer Characteristics and Cancer-Specific Mortality: An Overview. World J. Mens. Health.

[35] Zeng H., Xu M., Xie Y., Nawrocki S., Morze J., Ran X., Shan T., Xia C., Wang Y., Lu L. (2023). Racial/ethnic disparities in the cause of death among patients with prostate cancer in the United States from 1995 to 2019: A population-based retrospective cohort study. eClinicalMedicine.

[36] Awasthi S., Berglund A., Abraham-Miranda J., Rounbehler R.J., Kensler K., Serna A., Vidal A., You S., Freeman M.R., Davicioni E. (2021). Comparative Genomics Reveals Distinct Immune-oncologic Pathways in African American Men with Prostate Cancer. Clin. Cancer Res..

[37] Deo S.H., Holwerda S.W., Keller D.M., Fadel P.J. (2015). Elevated peripheral blood mononuclear cell-derived superoxide production in healthy young black men. Am. J. Physiol. Heart Circ. Physiol..

[38] Feairheller D.L., Park J.Y., Sturgeon K.M., Williamson S.T., Diaz K.M., Veerabhadrappa P., Brown M.D. (2011). Racial differences in oxidative stress and inflammation: In vitro and in vivo. Clin. Transl. Sci..

[39] Liou G.Y., C’Lay-Pettis R., Kavuri S. (2024). Involvement of Reactive Oxygen Species in Prostate Cancer and Its Disparity in African Descendants. Int. J. Mol. Sci..

